# The First Case of Glyphosate Resistance in Johnsongrass (*Sorghum halepense* (L.) Pers.) in Europe

**DOI:** 10.3390/plants9030313

**Published:** 2020-03-03

**Authors:** Jose G. Vazquez-Garcia, Candelario Palma-Bautista, Antonia Maria Rojano-Delgado, Rafael De Prado, Julio Menendez

**Affiliations:** 1 Agricultural Chemistry and Soil Sciences, University of Córdoba, 14014 Cordoba, Spain; z82vagaj@uco.es (J.G.V.-G.); z82pabac@uco.es (C.P.-B.); arakidonis@hotmail.com (A.M.R.-D.); qe1pramr@uco.es (R.D.P.); 2 Departamento de Ciencias Agroforestales, Escuela Tecnica Superior de Ingenieria, Campus de La Rabida, Universidad de Huelva, Palos de la Frontera, 21819 Huelva, Spain

**Keywords:** translocation, metabolism, penetration, EPSPS, non-target site resistance, resistant weeds

## Abstract

Six Johnsongrass populations suspected of being glyphosate resistant were collected from railways and freeways near Cordoba (SW Spain), where glyphosate is the main weed control tool. The 50% reduction in shoot fresh weight (GR_50_) values obtained for these six populations ranged from 550.4 to 1169 g ae ha^−1^, which were 4.2 to 9 times greater than the value obtained for the susceptible population. Glyphosate was equally metabolized to the same extent in both resistant and susceptible populations, with no significant differences in either 5-enolpyruvylshikimate-3-phosphate synthase (EPSPS) inhibition or basal activity. No amino acid substitutions were observed in any of the resistant populations. Slight but significant differences in glyphosate penetration were observed among some but not all of the resistant populations and for the times of incubation assayed, although these differences were not considered further. The proposed primary mechanism of resistance in these six glyphosate-resistant Johnsongrass populations is reduced herbicide translocation, because the amount of glyphosate that translocated from treated leaves to shoots and roots in the susceptible population was double that observed in the resistant populations. As glyphosate multiple resistance due to more than one mechanism is not uncommon, this is the first time that glyphosate-resistant Johnsongrass populations have been fully described for all known mechanisms.

## 1. Introduction

Johnsongrass is a C4 perennial and rhizomatous grass weed native to the Mediterranean region with a current range area between 55° N and 45° S, which reproduces by seeds and rhizomes [1]. The vegetative propagation, high fecundity, seed dormancy, and residual seedbank life of Johnsongrass contribute to its weediness (reviewed in [2]), and make it one of the major weeds throughout the world [1], although its preeminence has been drastically downgraded with the use of the systemic herbicide glyphosate as the main weed-control tool for glyphosate-resistant crops [3].

Glyphosate is a broad-spectrum herbicide that exclusively acts via foliar uptake. The target site in plants is the inhibition of the enzyme 5-enolpyruvylshikimate-3-phosphate synthase (EPSPS) (EC 2.5.1.19), which catalyzes the conversion of shikimate-3-phosphate and phosphoenolpyruvate into 5-enolpyruvylshikimate-3-phosphate and inorganic phosphate in the shikimic acid pathway [4]. Inhibiting this enzyme prevents the biosynthesis of the aromatic amino acids phenylalanine, tyrosine, and tryptophan. Glyphosate has, for decades, been the leading and most widely used herbicide in agriculture [5]. The widespread adoption of glyphosate-resistant (GR) crop technology has been claimed to be one of the causes of its increasing use [6], although glyphosate has been extensively used in perennial crops and industrial areas as well. These industrial areas include path borders, railway lines, and recreation areas, where glyphosate has been used worldwide to control weeds [7]. Regardless of the cropping system, persistent reliance on glyphosate treatments as the exclusive weed management system has usually led to the evolution of glyphosate resistance in weeds [2], typically after 10 years of repeated applications [8]. Resistance to glyphosate has been classically ascribed to (a) reduced translocation of the herbicide, probably due to sequestration in the vacuole [9,10], (b) mutations in DNA coding sequences leading to an altered resistant form of EPSPS [11,12,13], (c) overexpression of EPSPS through gene amplification [14], and (d) metabolization of glyphosate into non-toxic compounds [15,16,17]. Other less frequent, but described, resistance factors are (a) reduced foliar retention of the herbicide [18] and (b) reduced absorption of the herbicide [19,20]. These mechanisms are present in the 317 glyphosate-resistant biotypes belonging to forty-nine weed species and subspecies already described. These species include 39 glyphosate-resistant cases in non-crop areas [21]. In addition, five Johnsongrass cases have been reported in Argentina and the USA, all of which are associated with GR crops [21]. Of those five cases, only two American cases have been partially described [20,22]. Therefore, the Argentinian biotypes showed reduced glyphosate translocation and leaf uptake, while the Arkansas biotype only showed limited translocation. However, the Arkansas plants were not EPSPS sequenced, and these two published cases were not tested for glyphosate metabolism and EPSPS overexpression. This is important since glyphosate multiple resistance due to more than one mechanism is not uncommon [15,23,24].

The goal of the present study was to fully describe the resistance to the non-selective herbicide glyphosate in several Johnsongrass weed populations currently infesting different rail and roadways in southern Spain.

## 2. Results and Discussion

### 2.1. Whole-Plant Dose-Response Assays

The herbicide rates causing both a 50% mortality (LD_50_) and 50% reduction in shoot fresh weight (GR_50_) compared to non-treated controls were calculated. Therefore, the obtained GR_50_ and LD_50_ values allow comparisons of the results to those of other resistant Johnsongrass populations, such as the Argentinian glyphosate-resistant Johnsongrass [2] and the glyphosate-resistant Johnsongrass from Arkansas [22]. While the susceptible (S) population showed similar [22] or slightly greater [2] dose-response values than those reported in previously described S populations, all the remaining six glyphosate-resistant populations (GR1 to GR6) tested were significantly resistant to glyphosate relative to the susceptible population in terms of both the effective dose growth reduction and mortality (Table 1). Both GR2 and GR5 were the most resistant populations, exhibiting resistance index (RI) values ranging from 10.96 (GR2, LD_50_) to 8.18 (GR5, GR_50_). Although they showed a lower degree of resistance than these two populations, GR1, GR3, GR4, and GR6 also displayed a significant level of resistance compared to S plants, with RI values ranging from 7.94 (GR4, LD_50_) to 4.23 (GR1, GR_50_). Under these conditions, we could split the six populations into two groups: the first one included the GR2 and GR5 populations, which were >10-fold (LD_50_) and >8-fold (GR_50_), respectively, more resistant compared to the S population. The second group included the rest of the populations, with GR_50_ and LD_50_ values lower than the thresholds set but still significantly different from the S values (Table 1).

The levels of resistance of both groups were similar to those of other Johnsongrass populations that have been described as highly resistant to glyphosate, such as the Arkansas [22] and Argentinian [2] populations. These populations are described as having an altered glyphosate translocation pattern [20,22], with this altered glyphosate translocation being responsible for the higher resistance. In addition, one of the Argentinian Johnsongrass populations showed reduced glyphosate leaf uptake [20].

### 2.2. EPSPS Basal Activity and Inhibition

**Figure 1:**
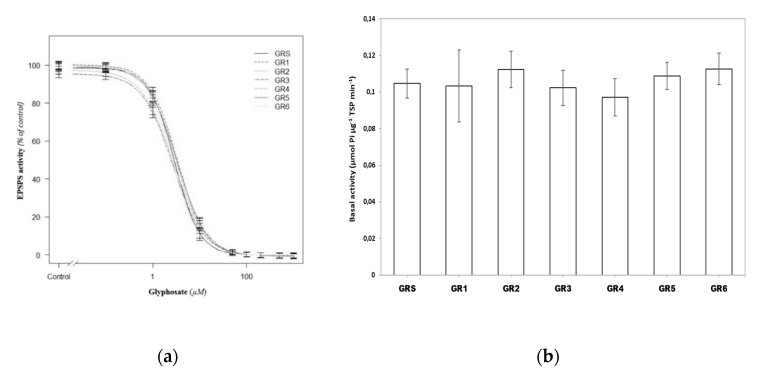
(**a**) 5-enolpyruvylshikimate-3-phosphate synthase (EPSPS) enzyme activity expressed as a percentage of the untreated control in leaf extracts of plants from resistant (GR) and susceptible (GS) populations of Johnsongrass. (**b**) Basal EPSPS activity, where histograms represent the treatment means (estimated in the absence of glyphosate) as vertical bars ± standard error (*n* = 6). No significant differences between resistant (R) and susceptible (S) populations were observed in both sets of data at α = 0.05.

Glyphosate target-site resistance (TSR) mechanisms have been associated with changes in EPSPS activity. These changes refer to both overexpression of the EPSPS gene associated with increased EPSPS gene amplification, EPSPS transcript levels, EPSPS protein expression, and/or genomic copy number, which increase its activity [14], or a mutation in the amino acidic sequence, which reduces its affinity for glyphosate binding [25]. In our case, there were no significant differences in terms of the EPSPS basal activity between the resistant and susceptible populations, with values ranging from 0.10 to 0.11 µmol phosphate µg total soluble protein (TSP)^−1^ (Figure 1). No additional data about *Sorghum halepense* EPSPS activity is available for comparison, but our values were similar to those observed in other glyphosate-susceptible EPSPS enzymes, such as those isolated from several *Conyza* species [26]. Therefore, even in a polyploidy species such as Johnsongrass, with multiple genes encoding the EPSPS protein, the overexpression of the EPSPS gene leading to multiple functional copies of the EPSPS protein does not seem to be the mechanism of resistance, as EPSPS basal activity remains the same no matter the biotype tested. In addition, there were no significant differences in the 50% inhibition of EPSPS activity (I_50_) values among all the populations tested, with estimated values ranging from 2.6 (GR6) to 3.4 (GR1) µM (Figure 1), which were even lower than those observed in other weed populations with glyphosate-susceptible EPSPS enzymes [26,27]. Therefore, a change of the sensitivity to glyphosate of the EPSPS enzyme is not the mechanism of resistance in the resistant populations.

### 2.3. EPSPS Gene Sequencing

TSR in glyphosate-resistant weed biotypes has been associated with amino acid substitutions at both the Thr102 and Pro106 positions of the EPSPS protein [28,29,30]. The partial sequence of the EPSPS2 gene revealed a similar sequence to those observed in glyphosate-resistant Argentinian populations [20], with no amino acid substitution at either the Pro106 or Thr102 positions in the glyphosate-resistant and -susceptible populations of Johnsongrass (Figure S1). These results, in conjunction with the patterns of EPSPS enzyme inhibition and basal activity, discard TSR mechanisms as the source of glyphosate resistance in resistant Johnsongrass populations.

### 2.4. Glyphosate Metabolism Study

The contribution of herbicide metabolism to non-target site resistance (NTSR) in glyphosate is somehow controversial. While some authors consider that this mechanism plays, at most, a minor role in glyphosate resistance [25,31], others claim this mechanism implies a decrease in the glyphosate concentration around its target site [32] or even changes in the translocation profile of glyphosate and its metabolites [16]. Glyphosate metabolism in plants is carried out by both a glyphosate oxidoreductase (GOX) putatively described as an aldo-keto reductase [17], which degrades the herbicide to glyoxylate and aminomethyl phosphonic acid (AMPA), and a carbon-phosphorus (C-P) lyase, which degrades glyphosate to sarcosine and inorganic phosphate, with formaldehyde present in that route as a reaction intermediate [31,33,34]. These two degradation pathways may be present or not in plants, although studies that include both degradation pathways are difficult and scarce [15,16]. Regardless of the metabolic pathway, glyphosate-tolerant legumes or weed species are characterized by a metabolic profile in which approximately 50% of the absorbed herbicide is degraded to other metabolites as fast as 96–144 h after treatment (HAT) [16,27] or even faster [17]. In our case, only AMPA and glyoxylate metabolites related to the GOX pathway were detected in both the resistant and susceptible Johnsongrass plants (Table 2). 

This result does not mean there is a lack of C-P lyase activity, because this metabolic pathway is slower than the GOX one and sarcosine is usually detected only at very long incubation times [16,27]. In any case, the tissular glyphosate levels at 120 HAT were quite similar and high in all of the populations assayed, with the AMPA levels also not significantly different (Table 2). The fact that the glyoxylate levels in the susceptible population were lower than those observed in some of the resistant ones (GR1, GR2, GR4, and GR5) does not seem relevant, because the differences are too small compared to those observed in metabolism-based NTSR cases. Because non-metabolized glyphosate accounted for 89.6 (GR3) to 91.3% (GR5) of the total glyphosate and its metabolites, our data discard metabolism as the mechanism of resistance.

### 2.5. Absorption and Translocation of ^14^C-Glyphosate

Unlike other susceptible and resistant Johnsongrass populations studied, in which glyphosate leaf absorption becomes asymptotic at only 24 HAT [20,22], glyphosate absorption in Spanish glyphosate-resistant and -susceptible plants was slower and more gradual, with the penetration percentages ranging from approximately 20% (24 HAT) to 80% (96 HAT and following) and the radioactivity recovered accounting for more than 90% of the applied ^14^C-glyphosate (Table 3). In these terms, there were differences in leaf absorption between the S biotype and five of the six resistant biotypes studied, although these differences were only statistically significant 24 and 48 HAT. No differences among populations were found at both 72 and 120 HAT, while only GR5 plants absorbed more glyphosate than S ones at 96 HAT (Table 3). Therefore, when comparing the two most extreme cases, even though susceptible plants absorbed 75.1% more glyphosate than the GR5 biotype 24 HAT, these differences were downgraded to 63.6% at 48 HAT and to non-significance with 22.8% and 11.8% 72 HAT and 120 HAT, respectively. Differences in glyphosate absorption as a source of herbicide resistance have been previously described in both glyphosate-resistant grass and broadleaved weeds, including one Argentinian glyphosate-resistant Johnsongrass biotype [20]. In our case, all the resistant biotypes had lower penetration values up to 72 HAT (Table 3), so a lower glyphosate leaf penetration as the mechanism of resistance would be plausible. However, we should consider these percentages carefully because differences in herbicide penetration do not always lead to resistance. In our case, although it is true that there were significant differences between the susceptible and resistant biotypes, it was also true that these differences faded over time, with all biotypes accumulating more than 74% of the recovered glyphosate 120 HAT. Whether the greater differences observed over shorter times of incubation or the subtle but significant differences observed at longer times made any difference in terms of glyphosate resistance is unknown because more than three-quarters of the glyphosate applied was present in all plants at the end of the experiment. Therefore, although it was very likely that glyphosate penetration played a role in the NTSR observed in our populations, the extent of this effect in the resistant response was not clear.

Likewise, there were clear differences in terms of glyphosate translocation between the susceptible and six resistant populations. The percentage of glyphosate remaining in the treated leaf 120 HAT ranged from 57.4% (GR2) to 70.4% (GR5) in resistant populations and decreased to 29.9% in the susceptible population (Table 4). This loss of herbicide in the susceptible treated leaves was found in both susceptible shoots and roots, with an accumulation of glyphosate that doubled, in most cases, that observed in resistant tissues (Table 4).

Thus, reduced glyphosate translocation to non-treated shoots and roots was the primary mechanism of resistance in our six glyphosate-resistant Johnsongrass populations. Altered translocation has been described as the main source of glyphosate NTSR in many broadleaved and grass weed biotypes, including the two American glyphosate-resistant Johnsongrass cases [20,22]. This mechanism seems to be associated with the glyphosate accumulation in the vacuole that led to reduced translocation [10]. In these terms, the activation in resistant (R)plants of two genes (M10 and M11) encoding two ATP-binding cassette transporters has been described as a putative explanation for low glyphosate transport in glyphosate-NTSR *Conyza Canadensis* (revised in [25]). The development of similar mechanisms of glyphosate resistance in three distant Johnsongrass locations raises interesting questions. When TSR both fails to provide an adequate level of resistance and increases the fitness penalty [35,36], as in the case of glyphosate, NTSR genes tend to accumulate until the fitness penalties associated with them make individuals unviable [37]. This pattern seems to be the case for cross-pollinated grass weeds, such as rigid ryegrass (*Lolium rigidum*), in which more than one different mechanism of resistance has often been described [35]. However, Johnsongrass has usually been described as a rather homozygous species with a natural tendency for clonal rhizome dispersion and self-pollination [38]. Although the percentage of outcrossing in Johnsongrass is being revised [39], the high degree of asexual reproduction may imply lower levels of genetic diversity. These lower levels of diversity may explain the presence of only one NTSR mechanism in our populations, a mechanism shared by all the glyphosate-resistant Johnsongrass populations described to date. Whether Johnsongrass is a weed species prone to developing certain mechanisms of resistance over others when exposed to glyphosate-mediated selection pressure or not, is an issue that requires further investigation.

## 3. Materials and Methods

### 3.1. Plant Material

A survey was conducted during June–July of 2015 and 2016 on Andalusian railways and roads starting from Cordoba city to other locations in the same region—Cordoba-Seville, Cordoba-Malaga, and Cordoba-Jaen. Next to these railways and roads are fruit and almond orchards, olive groves, and wheat and corn crop fields. Putative resistant (R) Johnsongrass seeds were collected from six different locations (GR1 to GR6 populations) and used for the assays described below. Seeds of a susceptible (S) Johnsongrass population were collected from a channel border that had not previously received glyphosate treatments near the laboratory facilities (Table S1).

All seeds were germinated in Petri dishes with filter paper moistened with distilled water and placed in a growth chamber at 28/18 °C (day/night) under a 16-h photoperiod, 850 µmol m^−2^ s^−1^ photosynthetic photon flux, and 80% relative humidity. Seedlings of the R and S populations were transplanted into pots containing sand and peat in a 1:1 (*v/v*) ratio and placed in a growth chamber under the environmental conditions described above.

### 3.2. Whole-Plant Dose-Response Assays

Glyphosate treatments were applied at the 3–4-leaf growth stage. Herbicide was applied within a laboratory spray chamber (SBS-060 De Vries Manufacturing, Hollandale, MN, USA) equipped with 8002 flat fan nozzles delivering 200 L ha^−1^ at 250 KPa at a height of 50 cm. The glyphosate (Roundup Energy^®^ SL, 450 g ae L^−1^, Monsanto Agricultura España, Madrid, Spain) rates ranged from 31.25 to 4000 g ae ha^−1^. After spraying, plants were maintained for 21 days in growth chambers under the previously described conditions. Afterward, R and S plants were cut at ground level, and growth was evaluated by plant mortality and aboveground fresh weight. The LD_50_ and GR_50_ rates were calculated for each experiment. The experiment was repeated twice and was arranged in a completely randomized design using five replicates (pots, four plants per pot) per rate.

### 3.3. EPSPS Inhibition and Basal Activity Study

EPSPS enzyme activity was studied according to Amaro-Blanco et al. [26]. Samples of 5 g of leaf tissue (three- to four-leaf growth stage) from each population were used for EPSPS extraction. The specific EPSPS activity in plants of each population was estimated in the presence and absence (basal activity) of glyphosate. EPSPS activity was determined using an EnzCheck Phosphate Assay Kit (Invitrogen, Carlsbad, CA, USA). The glyphosate concentrations used were 0, 0.1, 1, 10, 50, 100, 200, 500, and 1000 µM. Three replicates at each glyphosate concentration were used, and the experiment was repeated twice. The release of phosphate on the bottom level was measured over 10 min at 360 nm in a spectrophotometer (DU-640, Beckman Coulter, Inc., Fullerton, CA, USA). EPSPS enzyme activity was expressed as the percentage of enzyme activity in the presence of glyphosate with respect to the control (without glyphosate).

### 3.4. EPSPS Gene Sequencing

Total RNA was isolated from leaves using TRIzol reagent (Invitrogen, Carlsbad, CA, USA) according to the manufacturer’s instructions. RNA was then treated with TURBO DNase (RNase-Free; Ambion, Warrington, UK) to eliminate any DNA contamination and stored at −80 °C. cDNA synthesis was carried out from 2 μg of total RNA using the Moloney Murine Leukemia Virus (M-MLV) Reverse Transcriptase (Invitrogen, Carlsbad, CA, USA) in combination with oligo (dT)12–18 and random nonamers (Amersham Biosciences, Amersham, UK) according to the manufacturer’s instructions. To amplify the EPSPS gene, primers previously designed by Perez-Jones et al. [30] (forward: 5′ AGCTGTAGTCGTTGGCTGTG 3′; reverse: 5′ GCCAAGAAATAGCTCGCACT 3′) were used. These primers amplify a 543-bp fragment of the EPSPS gene that contains the mutation site that has been described to confer resistance to glyphosate in *Lolium* spp. [12,13]. Polymerase Chain Reaction (PCR) was carried out using cDNA from 50 ng of total RNA, 1.5 mM MgCl_2_, 0.2 mMdNTP, 0.2 μM of each primer, 1x buffer, and 0.625 units of a 100:1 enzyme mixture of non-proofreading (Thermusthermophilus) and proofreading (Pyrococcusfuriosus) polymerases (BIOTOOLS, Madrid, Spain) in a final volume of 25 µL. All PCR reactions were performed in duplicate, and the cycling conditions were 94 °C, 3 min; 35 cycles of 94 °C, 30 s; 55 °C, 30 s; and 72 °C, 1 min; and a final extension cycle of 72 °C, 10 min. An aliquot of the PCR product was loaded onto a 1% agarose gel to check for correct band amplification. The rest of the PCR products were purified using ExoSAP-IT^®^ for PCR Product Cleanup (USB, Ohio, OH, USA) as indicated by the manufacturers. Five purified PCR products per biotype were sequenced (STAB VIDA, Caparica, Portugal). Finally, the EPSPS DNA and the predicted peptide sequences were searched in the GenBank database using basic local alignment search tool (BLAST) on the website (http://www.ncbi.nlm.nih.gov/BLAST.cgi). The EPSPS sequences used for comparison were Argentinian glyphosate-resistant (HQ436352.1) and -susceptible (HQ436354.1) Johnsongrass accessions and glyphosate-resistant *Lolium rigidum* (ACB05442).

### 3.5. Metabolism Study

Johnsongrass plants at the 3- to 4-leaf stage were treated at a glyphosate rate of 300 g ae ha^−1^ as described in the dose-response assays section, and other plants were kept without treatment as non-treated controls. At 120 h after treatment, following the methodology described by Rojano-Delgado et al. [40], leaf tissues were washed with distilled water, flash-frozen in liquid nitrogen, and stored at −40 °C until use. Glyphosate and its metabolites, i.e., AMPA, glyoxylate, sarcosine, and formaldehyde, were determined by reversed-polarity capillary electrophoresis using a 3D Capillary Electrophoresis Agilent G1600A instrument equipped with a diode array detector (wavelength range 190–600 nm). The aqueous background electrolyte consisted of 10 mM potassium phthalate, 0.5 mM hexadecyltrimethylammonium bromide, and 10% acetonitrile at pH 7.5. The calibration equations were established from non-treated plants and known concentrations of glyphosate and its metabolites. The experiment was performed in a completely randomized design with six repetitions per biotype.

### 3.6. Absorption and Translocation of ^14^C-Glyphosate

To obtain the wetting agents and additives needed for absorption, ^14^C-glyphosate (American Radiolabeled Chemicals, Inc., Saint Louis, MO, USA) was added to the commercial herbicide to prepare a solution with a specific activity of 0.834 kBq µL^−1^. The final glyphosate concentration corresponded to 300 g ae ha^−1^ applied in 200 L ha^−1^. One 1 µL droplet was deposited by means of a micropipette (HTL LabMate) onto the adaxial surface of the second leaf of plants at the 3-leaf stage (0.834 kBq/plant). Preliminary assays with the populations studied here revealed that glyphosate absorption levelled off at 120 h after droplet application (results not shown). Three repetitions (considering every plant as a repetition) from each population were harvested at 24, 48, 72, 96, and 120 HAT. Treated leaves from each plant were carefully washed with 3 mL of a water‒acetone (1:1 *v/v*) solution to remove the unabsorbed ^14^C-glyphosate. The rinsate was analyzed by liquid scintillation spectrometry (LSS) on a Beckman LS 6500 scintillation counter. The remainder of the plant was carefully removed from the pot, and its roots were carefully washed with distilled water. Plants for penetration studies were kept undivided, while plants for translocation studies were divided into treated leaves, remaining shoot tissue, and roots. The plant parts obtained were dried at 60 °C for 96 h and combusted in a Packard Tri Carb 307 biological sample oxidizer. Evolved ^14^CO_2_ was trapped and counted by LSS in an 18 mL mixture of Carbo-Sorb E and Permafluor E+ (1:1 *v/v*) (Perkin-Elmer, Packard Bioscience BV). Foliar absorption (%) was calculated as (radioactivity recovered from plant parts)/(total radioactivity recovered) × 100. Translocation (%) was calculated as (total radioactivity in treated leaf, shoot or root)/(total radioactivity in all tissues) × 100. The amount of radiolabel deposited was determined by washing a treated leaf excised immediately after deposition (three replicates). The mean (SE) radioactivity recoveries ranged from 92% (6.3) to 94% (2.7) for resistant and susceptible Johnsongrass populations, respectively.

### 3.7. Statistical Analysis

Dose-response and EPSPS enzyme activity data were subjected to non-linear regression analysis using a three-parameter log-logistic model (Equation (1)) to determine the glyphosate dose that resulted in a 50% reduction in growth (GR_50_), 50% reduction in mortality (LD_50_), or 50% inhibition of EPSPS activity (I_50_).

Y = d/[1 + (x/g)^b^] (1)

where Y is the reduction of aboveground fresh weight, survival, or enzyme activity, expressed as the percentage of that of the non-treated control; d is the coefficient corresponding to the upper asymptote; b is the slope around the inflection point; g is the herbicide rate at the halfway inflection point (GR_50_, LD_50_, I_50_); and x (independent variable) is the herbicide rate.

Regression analysis was conducted using the statistical freeware program R 3.2.4 with the drc package [41]. Resistance indexes (RI) were calculated as R-to-S GR_50_, LD_50_, or I_50_, values. The values of GR, LD, and I were considered significantly different when their respective RI ratios differed from 1 at α = 0.05. Data from shikimate, basal enzyme activity, metabolism, penetration, and translocation studies were subjected to analysis of variance (ANOVA), and the Tukey honestly significant difference test was used to separate population means when needed. For cases of replicated whole experiments, ANOVA was conducted according to a generalized randomized block design, with each run of the experiment representing a block. In neither case did the interaction terms include block significance, and thus, they were not included in the final ANOVA models. Model assumptions of a normal distribution of errors and homogeneity of variance were checked by visual inspection of the residual plots. When required, data were square-root transformed. In those few cases, the non-transformed values are presented for clarity.

## Figures and Tables

**Table 1:** Parameters of the log-logistic equations used to calculate the glyphosate rates required for 50% reductions in fresh weight (GR_50_) and percent survival (LD_50_) expressed as the percentage of the mean untreated control of the Johnsongrass populations.

Survival (%)						Fresh Weight Reduction (%)				
Population	d	b	LD_50_ (g ae ha^−1^) ^a^	RI ^b^	*p*	d	b	GR_50_ (g ae ha^−1^) ^a^	RI ^b^	*p*
GR1	97.31	3.18	1588.95 ± 101.12	7.69	<0.0001	102.09	1.12	550.36 ± 43.85	4.23	<0.0001
GR2	99.83	3.19	2265.54 ± 56.63	10.96	<0.0001	102.53	1.53	1169.25 ± 57.49	8.99	<0.0001
GR3	100.91	2.64	1410.91 ± 68.25	6.83	<0.0001	101.15	1.48	959.02 ± 60.35	7.37	<0.0001
GR4	100.10	2.09	1640.36 ± 40.13	7.94	<0.0001	100.88	1.33	933.46 ± 24.17	7.17	<0.0001
GR5	100.73	2.49	2121.83 ± 120.06	10.27	<0.0001	101.81	1.59	1064.00 ± 113.53	8.18	<0.0001
GR6	99.97	2.41	1448.68 ± 62.04	7.01	<0.0001	100.07	1.11	751.30 ± 59.75	5.77	<0.0001
GS	100.07	4.45	206.55 ± 2.85	-		102.73	1.53	130.02 ± 13.59	-	

^a^ Mean values (*n* = 10) ± S.E. LD_50_: glyphosate rate needed to increase mortality by 50%, GR_50_: glyphosate rate needed to reduce fresh weight by 50%, ^b^ RI (Resistance Index): GR_50_ or LD_50_ (R)/GR_50_ or LD_50_ (S).

**Table 2:** Metabolism of glyphosate at 120 h after treatment in glyphosate-resistant (GR1 to GR6) and -susceptible (GS) Johnsongrass populations. Plants were treated with 300 g ae ha^−1^ glyphosate at the 3–4 leaf stage.

	% Recovered Metabolites ^a^		
Population	Glyphosate	AMPA	Glyoxylate
GR1	89.7 ± 3.9	8.2 ± 1.3ab	2.1 ± 0.8a
GR2	90.5 ± 2.3	7.5 ± 2.6ab	2.0 ± 0.4a
GR3	89.6 ± 4.2	9.1 ± 2.2a	1.3 ± 0.7bc
GR4	90.2 ± 2.4	8.0 ± 0.9ab	1.8 ± 0.3ab
GR5	91.3 ± 3.2	6.4 ± 2.1b	2.3 ± 0.4a
GR6	90.0 ± 3.9	8.6 ± 1.8a	1.4 ± 0.3bc
GS	91.1 ± 2.7	7.9 ± 0.9ab	1.0 ± 0.3c
*p*-value	0.1239	0.0089	0.0001

^a^ Data are the means of six repetitions ± S.E. Means with the same letters within a column are not significantly different at α = 0.05 based on Duncan’s post hoc test. AMPA: aminomethyl phosphonic acid.

**Table 3:** ^14^C glyphosate uptake in resistant (GR1 to GR6) and susceptible (GS) Johnsongrass populations at different hours after treatment (HAT).

	% of Applied Label ^a^				
Population	24 HAT	48 HAT	72 HAT	96 HAT	120 HAT
GR1	18.3 ± 1.5a	39.8 ± 3.0a	72.4 ± 4.5a	80.1 ± 6.9ab	84.2 ± 5.0a
GR2	20.8 ± 4.0a	40.3 ± 3.2a	70.3 ± 6.1a	80.4 ± 4.1ab	78.9 ± 1.7a
GR3	17.4 ± 2.8a	42.6 ± 4.9a	71.8 ± 5.5a	78.9 ± 5.9ab	85.3 ± 3.8a
GR4	22.7 ± 2.5ab	50.1 ± 5.4ab	70.3 ± 4.2a	81.6 ± 4.1ab	82.4 ± 3.0a
GR5	17.3 ± 1.7a	36.8 ± 3.8a	62.6 ± 3.1a	70.3 ± 3.1a	74.8 ± 4.1a
GR6	21.2 ± 2.7a	40.4 ± 5.1a	68.4 ± 3.9a	78.7 ± 2.9ab	82.6 ± 4.8a
GS	30.3 ± 2.5b	60.2 ± 6.7b	76.9 ± 3.9a	86.4 ± 4.8b	83.6 ± 5.3a

^a^ Data are the means of six repetitions ± S.E. Means with the same letters within a column are not significantly different at α = 0.05 based on Duncan’s post hoc test.

**Table 4:** ^14^C glyphosate translocation in the resistant (GR1 to GR6) and susceptible (GS) Johnsongrass populations at 120 h after treatment.

	% of Absorbed Label ^a^		
Population	Treated Leaf	Shoot	Root
GR1	60.8 ± 4.2c	24.6 ± 4.1b	14.6 ± 2.9d
GR2	57.4 ± 8.2c	23.1 ± 2.9b	19.5 ± 4.1b
GR3	62.8 ± 6.3bc	20.8 ± 3.2c	16.4 ± 1.8cd
GR4	70.1 ± 2.9a	15.8 ± 3.2d	14.1 ± 2.4d
GR5	70.4 ± 6.9a	15.8 ± 2.7d	13.8 ± 4.5d
GR6	67.9 ± 4.7ab	13.4 ± 2.2e	18.7 ± 3.6bc
GS	29.9 ± 2.8d	34.1 ± 2.1a	36.0 ± 3.9a

^a^ Data are the means of six repetitions ± S.E. Means with the same letters within a column are not significantly different at α = 0.05 based on Duncan’s post hoc test.

## Supplementary Materials

The following are available online at https://www.mdpi.com/2223-7747/9/3/313/s1: Figure S1: Partial sequences in the conservative region of 5-enolpyruvylshikimate-3-phosphate synthase (EPSPS) DNA isolated from both glyphosate-resistant (GR1 to GR6) and -susceptible (GRS) Johnsongrass populations; The EPSPS sequences used for comparison were glyphosate-resistant *Sorghum halepense* (HQ436352.1), susceptible *Sorghum halepense* (HQ436354.1), and glyphosate-resistant *Lolium rigidum* (ACB05442); Table S1: Herbicide records and locations of the different Johnsongrass populations.
