# Supplementary material for: The First Case of Glyphosate Resistance in Johnsongrass (Sorghum halepense (L.) Pers.) in Europe
Source: Plants (Basel). 2020 Mar 3;9(3):313. doi: 10.3390/plants9030313 (PMC7154863; doi:10.3390/plants9030313)
Supplement: Supplementary file 1 [file plants-09-00313-s001.pdf]

Table S1. Herbicide records and locations of the different Johnsongrass populations.

Populations	GPS Coordinates	Non-Crop Area	Herbicide	Rates/Years[a]
GS	37.916998, -4.720426	Channel border	Mechanical control	-/-
GR1	37.608287, -4.627536	Railway	Glyphosate + 2,4-D	1080+500/10
GR2	37.801470, -4.808024	Freeway	Glyphosate +MCPA	1800+600/20
GR3	37.946214, -4.544852	Freeway	Glyphosate +MCPA	1800+600/20
GR4	37.814494, -4.896752	Local way	Glyphosate (2 applications/year)	1080+1800/15
GR5	37.796783, -5.002603	Local way	Glyphosate (>2 applications/year)	1800+1800/>20
GR6	37.603694, -4.628106	Railway	Glyphosate + 2,4-D	1080+500/10

[a]Herbicide rates are expressed in g ae ha^{-1} (glyphosate) or g ai ha^{-1} (auxinic herbicides).

Amino acid location	99	100	101	102	103	104	105	106	107	108
GR1	N	A	G	T	A	M	R	P	L	T
GR2	N	A	G	T	A	M	R	P	L	T
GR3	N	A	G	T	A	M	R	P	L	T
GR4	N	A	G	T	A	M	R	P	L	T
GR5	N	A	G	T	A	M	R	P	L	T
GR6	N	A	G	T	A	M	R	P	L	T
GRS	N	A	G	T	A	M	R	P	L	T
HQ436352.1 (R)	N	A	G	T	A	M	R	P	L	T
HQ436354.1 (S)	N	A	G	T	A	M	R	P	L	T
ACB05442 (R)	N	A	G	T	A	M	R	S	L	T

Figure S1. Partial sequences in the conservative region of 5-enolpyruvylshikimate-3-phosphate synthase (EPSPS) DNA isolated from both glyphosate-resistant (GR1 to GR6) and -susceptible (GRS) Johnsongrass populations The EPSPS sequences used for comparison were glyphosate-resistant *Sorghum halepense* (HQ436352.1), susceptible *Sorghum halepense* (HQ436354.1), and glyphosate-resistant *Lolium rigidum* (ACB05442).
